# Pathological Tendon Histology in Early and Chronic Human Patellar Tendinopathy

**DOI:** 10.1155/2022/2799665

**Published:** 2022-10-04

**Authors:** Nikolaj Moelkjaer Malmgaard-Clausen, Michael Kjaer, Stephanie G Dakin

**Affiliations:** ^1^Institute of Sports Medicine Copenhagen, Department of Orthopaedic Surgery, Copenhagen University Hospital-Bispebjerg and Frederiksberg, Copenhagen, Denmark; ^2^Center for Healthy Aging, Department of Clinical Medicine, University of Copenhagen, Copenhagen, Denmark; ^3^Nuffield Department of Orthopaedics, Rheumatology and Musculoskeletal Sciences (NDORMS), University of Oxford, Oxford, UK

## Abstract

The present pilot study investigated the extent of histological tissue changes in both chronic tendinopathy and in individuals that display early clinical signs of tendinopathy. The study included 8 individuals of whom 3 were healthy without any tendon symptoms, 2 had early symptoms (1–2 months), and 3 had chronic symptoms (>3 months) from their patellar tendons. Percutaneous needle biopsy samples were obtained from the affected tendon tissue region. Biopsy samples were stained with Haematoxylin & Eosin, and multiplex immunofluorescence staining was performed for markers of inflammation and resolution. Both early and chronic stage patellar tendon biopsy samples from this small patient cohort exhibited expansion of the interfascicular matrix (IFM) and endotenon regions together with increased cellularity and vascularity. These histological observations were moderate in early tendinopathy, whereas they were more pronounced and associated with marked disruption of tissue architecture in chronic tendinopathy. Early stage tendinopathic patellar tendons expressed markers associated with an activated phenotype of fibroblasts (CD90, CD34), macrophages (S100A8), and endothelial cells (ICAM1, VCAM1). These tissues also expressed enzymes implicated in inflammation (PTGS2, 15PGDH) and resolution (ALOX12) and the proresolving receptor ERV1. Immunopositive staining for these markers was predominantly located in the IFM regions. These preliminary findings suggest that mild to moderate structural histological changes including expansion of IFM and endotenon regions are pathological features of early tendinopathy, and support inflammatory and resolving processes are active in early-stage disease. Further investigation of the cellular and molecular basis of early-stage tendinopathy is required to inform therapeutic strategies that prevent the development of irreversible chronic tendon disease.

## 1. Introduction

Tendon overuse disease in the form of tendinopathy is a common disorder in sports activity, leisure activity, or occupational activity and will impair performance and occupational capacity [1] and affects up to 30% of the entire population [2]. Chronic tendinopathy (symptom duration > 3 months) is associated with morphological changes in the form of increased tendon size, increased vascularization and water content of the tendon, areas with disorganized fibril structure, rounded fibroblast morphology, and upregulation and increased protein content of several matrix proteins [3–7]. Despite these documented changes in structure and protein profile in pathological tendon, less is known of the histopathological features of early-stage tendinopathy (<3 months duration) and the precise pathobiology during this stage of disease.

Expression of inflammatory biomarkers and increased cellular infiltration have been demonstrated in human tendon disease. Furthermore, it has been demonstrated that stromal fibroblast activation is a feature of chronic tendinopathy in the shoulder and Achilles [8, 9] and that macrophages exhibit a complex phenotype, which alters with disease stage [9]. In further support of this inflammatory milieu, diseased tendon tissues also express alarmins including HIF-1alpha, S100 proteins, IL-33, and HMGB1 [10–15]. These molecules are potentially important for regulation of acute inflammation and resolution [15]. Recently, a multiomics single cell analysis of diseased and healthy human tendons revealed multiple subpopulations of tendon cells expressing matrix associated genes [16]. This study demonstrated that chronic tendinopathy was coupled to an increased expression of proinflammatory markers in microfibril-associated tenocytes and that vascular endothelial cells in diseased tendons demonstrated increased expression of chemokines and alarmin genes. Despite the fact that inflammation appears to be an important driver in tendon disease, there is limited evidence on the effect of classical oral nonsteroidal anti-inflammatory drugs (NSAIDs) both in early and chronic tendinopathy [17, 18].

Previous results indicate that at least some of the changes at the cellular level are initiated in overloaded tendons even before the onset of pain [19]. Therefore, it is likely, but not proven, that a broad profile of molecular and cellular changes is present in tendinopathy already at onset of symptoms. Contrary, in a recent study on early Achilles or patellar tendinopathy, only marginal altered mRNA signalling for tendon growth was observed [20], which could indicate that biochemical changes may not be an early phenomenon, but this is not known.

The present pilot study investigated the extent of histological tissue changes in both chronic tendinopathy and in individuals that display early onset of clinical signs of tendinopathy. We hypothesized that the histopathological changes would be profound in chronic tendinopathy and that early-stage injured tendons would express markers of inflammation and resolution.

## 2. Materials and Methods

### 2.1. Collection of Patellar Tendon Biopsies

Participants were included from a larger cross-sectional study including 45 study participants and investigating clinical, imaging, and histological features of tendinopathy at different stages of disease. The small subset of participants included in the current study were consecutively included in the initial phase of the recruitment process. This was chosen for practical reasons since the analytical setup was only available for a limited period. For the current study, we aimed to include bilateral patellar tendon biopsies from 9 sports active participants; however, since the analytical setup was only available for a limited period, 8 participants were included. Of these participants, three were healthy controls (control (CTRL)), two were patients with patellar tendinopathy and a symptom duration from 1–60 days (early tendinopathy (ET)), and three subjects were patients with patellar tendinopathy and a symptom duration ≥90 days (chronic tendinopathy (CT)). Subjects were eligible if they had no concomitant injuries or diseases, were 18–45 years old, and had a BMI from 18.5–30. In the two patient groups, the diagnosis was established through the medical history and a clinical examination by an educated physician. Furthermore, the diagnosis was confirmed using greyscale and power Doppler ultrasonography. Symptomatic study participants were found eligible if they had a relevant history of activity-related pain in the patellar tendon, palpation pain in the proximal part of the patellar tendon, and changes on ultrasound including hypoechogenicity, increased power doppler signal, or increased anterior-posterior diameter. Exclusion criteria included previous knee surgery, previous injections, smoking, arthritis, diabetes, hypercholesterolemia, and MRI contraindications. The ET and CTRL group subjects were not allowed to have had any previous injuries in the patellar tendons. On the initial visit, subjects were further asked to answer questionnaires concerning the amount of weekly physical activity and symptom severity. Bilateral percutaneous patellar tendon biopsies were performed on a separate visit under sterile conditions using a 14-G automatic biopsy needle (Bard Magnum Biopsy Instrument; CR Bard, Covington, GA). Subjects were lying prone with the knee joint in 90 degree-flexion, hair was removed from the area, and the overlying skin was anesthetized with 2 ml of lidocaine (1%). The area was sterilized, and a 5 mm incision was made in the skin to expose the tendon. The biopsies were obtained in the most severely affected area, the tendon, and was planned based on ultrasonography scans; in healthy individuals, the biopsies were obtained in the proximal central midtendon. Since we were primarily interested in the direct comparison between healthy subjects and symptomatic subjects, and furthermore, because of the challenging nature of the samples, we chose to only include analyses of the symptomatic side in subjects with patellar tendinopathy. The study was approved by the regional ethical committee Danish Regional Ethical Committees of the Capital Region (H-18048248) and the Danish Data Protection Agency (VD-2019-150).

### 2.2. Tissue Processing of Collected Tendon Biopsies

Patella tendon biopsies were collected in 10% of formalin and stored at room temperature for 1 week. Tissues were then transferred into 70% of EtOH and stored at 4°C. Samples were processed in a Leica ASP300S tissue processor and subsequently embedded in paraffin wax; 6 mm tissue sections were cut using a RM2135 microtome (Leica Microsystems) and baked onto adhesive glass slides at 60°C for 30 min and 37°C for 60 min. Tissue sections were stained with Haematoxylin & Eosin using established protocols and slides mounted in the DPX mountant (Fischer Scientific). Stained slides were imaged using an automated slide scanner (MOTIC).

### 2.3. Multiplex Immunostaining of Patellar Tendon Biopsies

Multiplex immunostaining of patellar tendon tissues was performed using a previously established protocol [9]. After deparaffinization, antigen retrieval tissues were blocked in 5% of normal goat serum (Sigma) in phosphate-buffered saline (PBS) for 60 min in a humid chamber at room temperature. Sections were incubated with the primary antibody cocktail diluted in 5% of normal goat serum in PBS for 2.5 hours at room temperature. The details of primary antibodies used for immunofluorescence are listed in Table S1. Sections were washed 3 times with PBS-Tween 20 (PBST) for 5 min. Slides were incubated in the secondary antibody cocktail, each diluted 1 : 200 in 5% of normal equine serum (Sigma) in PBS for 2.5 hours and shielded from light. Secondary antibodies were Alexa Fluor goat antimouse IgG2a or IgG2b or goat antirabbit IgG (Life Technologies) and goat antimouse IgG_1_ (Southern Biotech). After washing, sections were incubated in 2 mM POPO-1 nuclear counterstain (Life Technologies) and diluted in PBS containing 0.05% saponin (Sigma) for 20 min. Tissue auto fluorescence was quenched with a solution of 0.1% Sudan Black B (AppliChem) in 70% ethanol for 10 min. Slides were mounted using fluorescent mounting medium (VectaShield), sealed, and stored at 4°C until image acquisition. For immunostaining controls, the primary antibody was substituted for universal isotype control antibodies: cocktail of mouse IgG_1_, IgG_2a_, IgG_2_b, IgG_3_, and IgM (Dako) and rabbit immunoglobulin fraction of serum from nonimmunized rabbits, solid-phase absorbed (Dako). Isotype control images are shown in Supplementary Figure 1. Images were acquired on a Zeiss LSM 710 con-focal microscope using a 40× oil immersion objective (numerical aperture, 0.95). The fluorophores POPO-1, Alexa Fluor 488, Alexa Fluor 568, and Alexa Fluor 633 were excited using the 405-, 488-, 561-, and 633-nm laser lines, respectively. Two-dimensional image reconstructions were created using ZEN (Zeiss).

## 3. Results

### 3.1. Participant Characteristics

Study participant characteristics are presented in Table 1. All study participants were sports active individuals with a mean weekly weight bearing activity level of 5.3 h/week. Subjects were [21–39] years old. The ET patients had symptom duration [54–58 days], whereas the CT patients had symptom duration from [165–670 days]. The numerical ranking scale (NRS) for pain during activity was between [3–4] for ET patients and between [4–9] for CT patients, and NRS for pain in the single leg decline squat test was between [1–4] and [5–5] for ET and CT, respectively. All subjects participated in weight bearing activities, although this was markedly reduced for the patient groups after the onset of injury.

### 3.2. Histopathological Features of Early-Stage Patellar Tendinopathy

**Figure 1:**
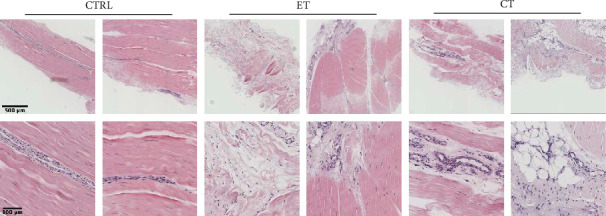
Representative longitudinal histological sections stained with Haematoxylin & Eosin. Top row low magnification (scalebar: 500 *µ*m) and bottom row high magnification (scalebar: 100 *µ*m). 2 biopsies from each group are displayed (healthy controls (CTRL), patients with early tendinopathy (ET) and patients with chronic tendinopathy (CT)).

H&E staining revealed a disturbed architecture in both the early and chronic tendinopathy tissues when compared to healthy tissue sections. Notable pathological features in diseased sections included hypercellularity, rounding of tendon cells, expansion of the IFM and endotenon regions, loss of tissue integrity, and increased vascularity. These observed changes appeared more subtle in early tendinopathy tissue sections. Chronically diseased patellar tendons exhibited almost complete loss of seemingly healthy IFM with significant distortion of the tissue architecture, contrasting markedly with healthy tendons (Figure 1).

### 3.3. Multiplex Immunostaining for Markers of Tendon Pathology

**Figure 2:**
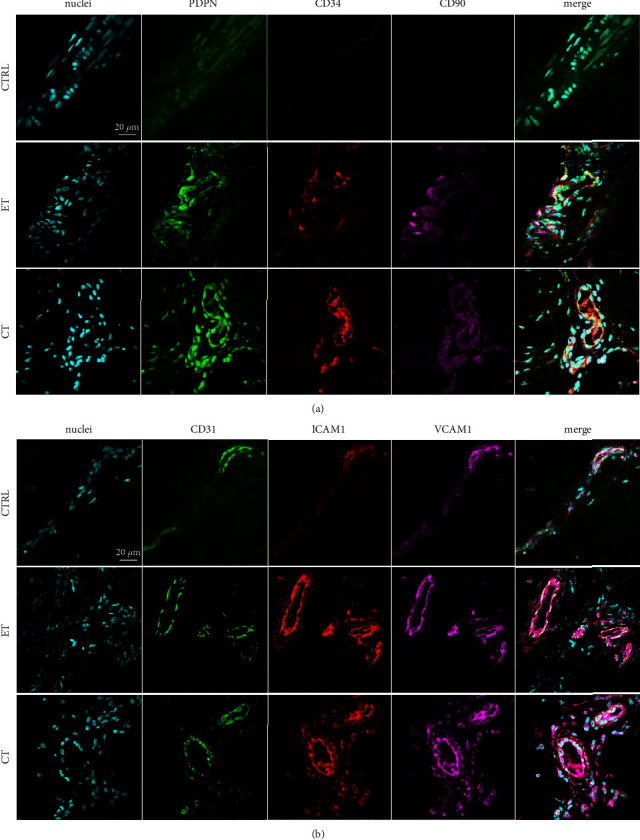
Multiplex immunostained longitudinal patellar tendon biopsy sections from healthy controls (CTRL), patients with early tendinopathy (ET), and patients with chronic tendinopathy (CT). Scalebar: 20 *µ*m. (a) It shows increase for PDPN (green), CD34 (red), and CD90 (purple). (b) It shows staining for CD31 (green), ICAM1 (red), and VCAM1 (purple). Nuclei (blue in 2(a) and 2(b)).

In diseased musculoskeletal tissues, fibroblasts, macrophages, and endothelial cells are known to express an activated phenotype [21–23]. We therefore investigated if markers of these processes were present in early-stage patellar tendinopathy. Multiplex immunostaining for fibroblast activation markers (PDPN/CD34/CD90) revealed very little staining in healthy tendon (Figure 2(a)). These markers were expressed in both early and chronic tendinopathy. ET staining of fibroblast activation markers was primarily located at the intrafascicular matrix, whereas staining was more diffuse in chronic tendinopathy sections and particularly located at the perivascular regions (Figure 2(a)).

**Figure 3:**
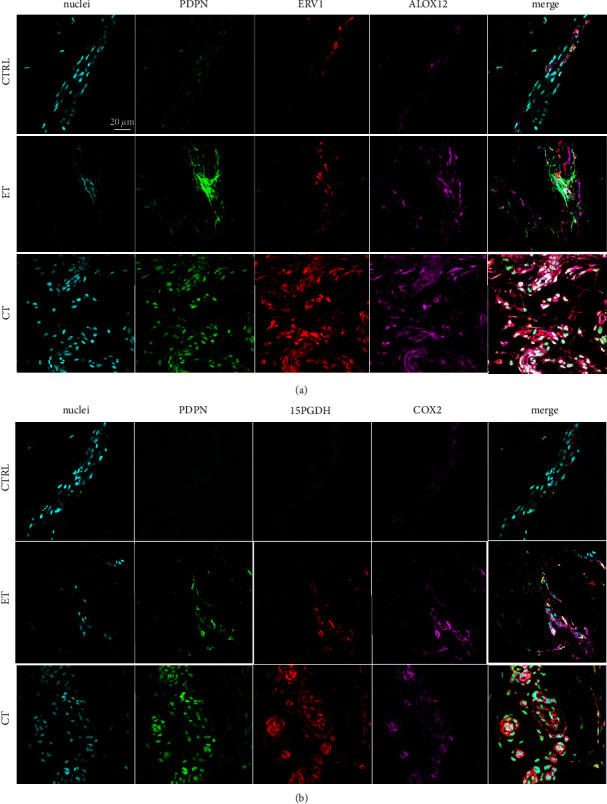
Multiplex immunostained longitudinal patellar tendon biopsy sections from healthy controls (CTRL), patients with early tendinopathy (ET), and patients with chronic tendinopathy (CT). Scalebar: 20 *µ*m. (a) It shows staining for PDPN (green), ERV1 (red), and ALOX12 (purple). (b) It shows staining for PDPN (green), 15PGDH (red), and COX2 (purple). Nuclei (blue in 3(a) and 3(b)).

**Figure 4:**
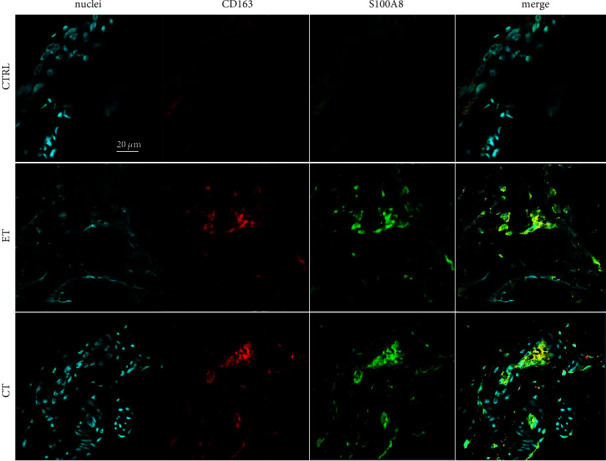
Multiplex immunostained longitudinal patellar tendon biopsy sections from healthy controls (CTRL), patients with early tendinopathy (ET), and patients with chronic tendinopathy (CT). Scalebar: 20 *µ*m. Nuclei (blue), CD163 (red), and S100A8 (green).

Both early and chronic stage tendinopathy tissues expressed markers of vascular endothelial activation (CD31 ICAM1 VCAM1) relative to healthy tendons. Vessels were generally localized to IFM and endotenon regions (Figure 2(b)). Furthermore, immunostaining for markers of enzymes implicated in inflammation (PDPN/15PGDH/COX2) and receptors and enzymes implicated in resolution (PDPN/ERV1/ALOX12) revealed expression of these proteins in early tendinopathy (Figures 3(a) and 3(b)). These markers were abundantly expressed in chronic disease, with minimal expression in healthy tissues (Figures 3(a) and 3(b)). We identified S100A8 immunopositive staining which colocalized with the macrophage marker CD163 in early-stage tendinopathy. Healthy tissues showed very low-level expression of these markers, in contrast to chronic tendinopathy that showed profound expression of this inflammatory macrophage phenotype (Figure 4). It was more challenging to locate the precise topographical expression of these markers in chronic stage disease due to the significant distortion of the tendon structure. In early-stage disease, these markers of inflammation and resolution could be mapped to IFM and endotenon regions which showed mild expansion but retained their topographical architecture.

## 4. Discussion

The present study is a small pilot study to observe immunohistochemical differences between healthy, early phase tendinopathy and later stage chronic tendinopathy in human patellar tendon. We demonstrated that moderate expansion of the IFM and the endotenon regions together with an increased cellularity and vascularity are histopathological features of early tendinopathy. These structural changes appear to be more marked in chronic tendinopathy where tissues also exhibit significant disruption to the tissue architecture. We further show that early tendinopathy tissues exhibit markers of fibroblast, macrophage, and vascular endothelial activation and propose that inflammatory and resolving processes may occur in IFM and endotenon topographical niches in early-stage disease.

It is well known that tissue architecture is disrupted in chronic tendinopathy, and there have been similar findings in early tendinopathy [4, 24]. However, no direct comparisons have been made within the same tendon at different stages of disease. In the current work, a direct comparison was made between patellar tendons from healthy controls with no previous history of tendinopathy and patellar tendons from patients at two distinct stages of disease. This enables us to rule out potential differences between tendons at different locations in the body. In the current study, we observed a less pronounced disruption of the tissue architecture in patients with early tendinopathy compared to patients with chronic tendinopathy, whereas changes in early tendinopathy mainly occurred in the IFM, changes were more profound in chronic tendinopathy and involved the fascicular matrix. However, a clear difference was observed between early tendinopathy and healthy control tissues. Thus, structural changes especially located to the IFM may precede the onset of symptoms.

Together with the well-known histological features, inflammation has been established as a key feature in tendinopathy [23]. Furthermore, it has been proposed that the degree and character of inflammation changes during disease progression [19]. The current results confirm that inflammation is a sustained feature of tendinopathy long time after the onset of symptoms. However, no clear association with symptom duration was observed.

Markers of fibroblast activation, resolution, and vascularization are well-documented pathological features of chronic tendinopathy [8, 9, 25, 26]. Although not quantified, these markers generally showed lower-level expression in early tendinopathy compared to chronic tendinopathy. It is continuously discussed whether they are important for the development and progression of disease, nevertheless this study suggests that they are sustained features of tendinopathy [27].

The current study is exploratory in it its nature and included a very limited number of study participants, thus results cannot be generalized to the general population. However, the current findings in chronic tendinopathy are consistent with the previous findings which confirm applicability of the applied methods. Furthermore, to our knowledge, this is the first study to compare patellar tendon biopsies directly between healthy controls with no previous history of tendon disease and patients at different stages of tendon disease, which simplifies the interpretation of results. Since bilateral alterations are frequent in patients with unilateral symptoms, the contralateral biopsies in symptomatic participants were not used as control [28]. Furthermore, tendon biopsies are not suitable for longitudinal studies since the biopsy procedure itself has been shown to severely affect subsequent biopsies [29]. Biopsies from healthy individuals were all obtained in the proximal, central midtendon, whereas biopsies in symptomatic individuals were obtained in the most severely affected area of the tendon. We cannot exclude that this may have influenced our results. We do acknowledge that the limited tissue material obtained is not necessarily representative of the whole tendon; however, it would be unethical to obtain larger tendon biopsies in otherwise healthy young individuals. Furthermore, biopsies were planned based on ultrasound findings in symptomatic individuals and therefore considered representative for the tendinopathic part of the tendon. Moreover, the selected markers were primarily chosen based on our current understanding of chronic tendon disease; therefore, additional markers that may be of importance in early tendinopathy were not identified. We utilised known markers of fibroblast activation [21], inflammation, and resolution [9, 11] and vascular activation [8] previously established in the literature through the study of chronically diseased tendons. This was intentional as we wanted to ask the question if these biological processes could be identified in exercise-induced early-stage injury in patellar tendons obtained in this study. An unbiased approach (e.g. proteomics or scRNAseq) could have revealed important markers of early tendinopathy and thereby potential treatment targets; however tissue material was very limited, and cell numbers in especially healthy samples were very limited. Thus, the small size of the tendon samples precluded the use of unbiased approaches. Therefore, we chose to prioritize a single method approach in this exploratory study where spatial information of the tendon structure was preserved. Moreover, due to the very limited material available, we have omitted to include histological scoring of our samples since quantification of the observed changes would not lead to reliable statistical calculations. Future studies may benefit from more explorative approaches and should have larger sample size to enable reliable quantification of the observed changes. Lastly, the early tendinopathy model used in the current study is based on patient-reported symptom duration, and we cannot exclude and certainly expect tissue changes to precede onset of symptoms. Nevertheless, this population represents patients with shorter symptom duration and lower symptom burden, reflected in Table 1. Despite these limitations in this preliminary study, the current work still adds to our understanding of early tendon disease, emphasising that cellular and inflammatory changes in early-stage tendinopathy precede severe structural damage.

Collectively our results indicate that tendon tissue from patients with early tendinopathy appears less structurally disrupted compared to chronic tendinopathy, and expression of markers for inflammation, resolution, vascularization, and fibroblast activation are primarily located to the IFM. The profound tissue changes observed in chronic tendinopathy very likely explain why symptoms often persist in these patients [30]. In adult human tendons, the turnover of collagen is very limited, which suggests a limited capacity for tissue repair [31]. In the current study, the structural integrity of the fascicular matrix was better preserved in early tendinopathy, and thus, early stages of tendon disease may serve as a window of opportunity regarding treatment. A recent study in early Achilles tendinopathy results indicated that early targeted treatment leads to faster recovery. However, in the same study function remained impaired after 1 year. Thus, understanding the pathobiology of early tendinopathy is critical in enabling us to treat during an early therapeutic window before irreversible tissue changes occur with chronic disease.

These findings support the hypothesis that development of tendinopathy relates closely to pathological cellular and matrix changes which predominantly occur in the interfascicular region of human tendon. Further investigation of the distinct cell types and molecules active in early-stage tendinopathy is required to inform effective new therapeutic strategies that prevent the development of irreversible fibrotic chronic tendon disease.

## Figures and Tables

**Table 1:** Subject characteristics. Activity-pre: total weekly training time consumption, excluding nonweight-bearing activities, before injury in symptomatic study participants. Activity-current: current total weekly training time consumption, excluding nonweight-bearing activities. VISA-P: Victorian Institute of Sports Assessment-patellar questionnaire (score [0–100], 0 = lowest functional capacity; 100 = highest functional capacity). NRS: the numerical ranking scale during patellar loading activities (score [0–10], 0 = no pain; 10 = worst imaginable pain). Data are presented as mean (range). Healthy controls (CTRL), Early tendinopathy (ET), Chronic tendinopathy (CT).

	CTRL	ET	CT
Age (years)	23.7 (21–28)	29.5 (29–30)	30.7 (26–39)
Sex (m/f)	2/1	0/2	2/1
Weight (kg)	76.4 (70.8–82.1)	66.8 (64.2–69.4)	78.4 (72.3–84.4)
Height (cm)	172.4 (167.8–179.0)	166.8 (158.5–175.0)	178.5 (174.9–182)
Activity-pre (hrs/week)	—	6.5 (5–8)	7.7 (2–14)
Activity-current (hrs/week)	6.7 (3–12)	4.5 (3–6)	4.7 (2–9)
VISA-P	—	73.5 (72–75)	38.3 (30–54)
NRS	—	3.5 (3–4)	6.3 (4–9)
NRS-SLDS	—	2.5 (1–4)	5 (5–5)

## Data Availability

The authors confirm that the data supporting the findings of this study are available within the article and its supplementary material.

## Supplementary Materials

Supplementary Materials Supplementary Table 1: primary antibodies were used for immunofluorescence staining of human patellar tendon biopsy samples. Supplementary Figure 1: isotype control images for mouse and rabbit IgG antibodies, from longitudinal patellar tendon biopsy sections of chronic tendinopathy.
