# Supplementary material for: Pathological Tendon Histology in Early and Chronic Human Patellar Tendinopathy
Source: Transl Sports Med. 2022 Oct 4;2022:2799665. doi: 10.1155/2022/2799665 (PMC11022758; doi:10.1155/2022/2799665)
Supplement: Supplementary Materials — Supplementary Table 1: primary antibodies were used for immunofluorescence staining of human patellar tendon biopsy samples. Supplementary Figure 1: isotype control images for mouse and rabbit IgG antibodies, from longitudinal patellar tendon biopsy sections of chronic tendinopathy. [file 2799665.f1.zip › 2799665.f1/FigureS1.docx]

**
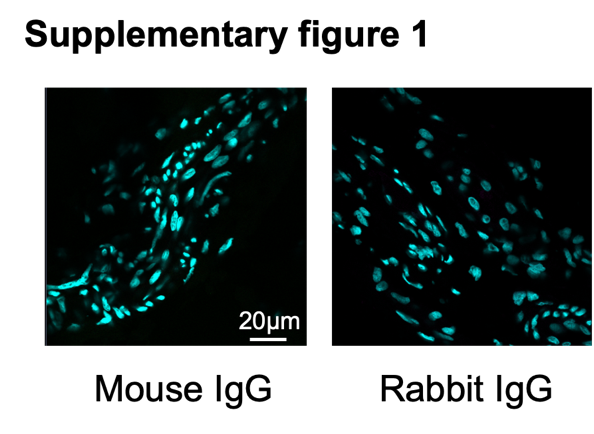
**

**Supplementary figure 1** Isotype control images for mouse and rabbit IgG antibodies, from longitudinal patellar tendon biopsy sections of chronic tendinopathy.
