# Supplementary material for: Pathological Tendon Histology in Early and Chronic Human Patellar Tendinopathy
Source: Transl Sports Med. 2022 Oct 4;2022:2799665. doi: 10.1155/2022/2799665 (PMC11022758; doi:10.1155/2022/2799665)
Supplement: Supplementary Materials — Supplementary Table 1: primary antibodies were used for immunofluorescence staining of human patellar tendon biopsy samples. Supplementary Figure 1: isotype control images for mouse and rabbit IgG antibodies, from longitudinal patellar tendon biopsy sections of chronic tendinopathy. [file 2799665.f1.zip › 2799665.f1/TableS1.docx]

**Table S1.** Primary antibodies used for immunofluorescence staining of human patellar tendon biopsy samples.

| **Antibody** | **Clone** | **Isotype** | **Species** | **Dilution** |
| --- | --- | --- | --- | --- |
| Podoplanin (PDPN)  Abcam  Ab10288 | 18H5 | IgG_1_ | mouse | 1:100 |
| CD90  Abcam  Ab92575 | [EPR3132] | IgG | rabbit | 1:200 |
| CD34  Abcam  Ab54208 |  | IgG2b | mouse | 1:200 |
| CD31  Abcam  Ab187377 | [C31.3] | IgG_1_ | mouse | 1:100 |
| CD106 (VCAM-1)  LS-Biosciences  LS_C313019 |  | IgG | rabbit | 1:100 |
| ICAM1  LS-Biosciences  Ab2213 | [MEM-111] | IgG2a | mouse | 1:200 |
| CD163  LS-Biosciences  LS_C174770 | 34B | IgG_2a_ | mouse | 1:150 |
| MRP8 (S100A8)  Abcam  Ab 180735 |  | IgG | rabbit | 1:200 |
| ERV1  Abcam  Ab168097 | 1A7 | IgG2b | mouse | 1:200 |
| ALOX12  Abcam  Ab211509 |  | IgG | rabbit | 1:200 |
| 15-PGDH  Abcam  Ab118185 |  | IgG2a | mouse | 1:200 |
| COX2  Abcam  Ab15191 |  | IgG | rabbit | 1:200 |

**Supplementary table 1** Primary antibodies used for immunofluorescence multiplex stainings (figure 2-4). Blue represents nuclear counterstain. Mouse isotypes IgG1, IgG2A and IgG2B.
